# An Account of a Singular Case of Ischuria, in a Young Woman, Which Continued for More Than Three Years; during Which Time, If Her Urine Was Not Drawn off with the Catheter, She Frequently Voided It by Vomiting; and, for the Last Twenty Months, Passed Much Gravel by the Catheter, as Well as by Vomiting, When the Use of That Instrument Was Omitted, or Unsuccessfully Applied. To Which Are Added Some Remarks and Physiological Observations

**Published:** 1795

**Authors:** Isaac Senter

**Affiliations:** Associate Member of the College of Physicians of Philadelphia, and Senior Surgeon in the Late American Army.


XIII. *An Account of a ſingular Caſe of Iſchuria, in a young Woman, which continued for more than three Years; during which Time, if her Urine was not drawn off with the Catheter, ſhe frequently voided it by vomiting; and, for the laſt twenty Months, paſſed much Gravel by the Catheter, as well as by vomiting, when the Uſe of that Inſtrument was omitted, or unſucceſsfully applied. To which are added ſome Remarks and Phyſiological Obſervations. By* Iſaac Senter, *M. D. Aſſociate Member of the College of Phyſicians of Philadelphia, and ſenior Surgeon in the late American Army.* Vide *Tranſactions of the College of Phyſicians, of Philadelphia. Vol.* I. *Part* I. 8vo. Philadelphia, 1793.

THE ſubject of this extraordinary caſe was a healthy-looking ſervant girl, who, in June, 1785, being then in her fifteenth year, was ſeized with a pain in the left hypochondrium, accompanied with cough, oppreſſion at her breaſt, dyſpnœa, and fever.

She had menſtruated pretty regularly from the age of thirteen till within five weeks of her preſent illneſs, which was aſcribed to cold.

Venæfection and other fuitable remedies were had recourfe to by Dr. Senter, to whom fhe applied for relief, and her complaints foon fubfided; but about a month afterwards fhe vomited up a quantity of bloody pus, which induced him to think a vomica had burft in her ftomach; for during the whole of this illnefs, her ftomach, it feems, was fo irritable, that fhe could with difficulty retain in it either food or medicine.

She had now a fuppreffion of urine, which, after continuing twenty-four hours, went off without any medical affiftance. After this fhe became regular in her menfes, and in about two months was fufficiently recovered to refume her employment as a fervant, which fhe continued to follow till the 3d of June, 1786, when all her former complaints (except the fuppreffion of the menfes) returned with greater feverity than before.

Her pulfe was now at 120; her ftomach, as during the former attack, was fo irritable, that fhe vomited up immediately almoft every thing fhe took. Of the different remedies that were had recourfe to, opium, when fhe could retain it on her ftomach, and repeated blood-letting in fmall quantities, gave her the moft relief.

On

On the 2d of July, when the ſeverity of the ſymptoms had ſubſided, ſhe was ſeized with a total ſuppreſſion of urine, which continued till the beginning of the ſixth day, when a vomiting came on, which laſted till ſhe brought up nothing but water; and this water, ſhe ſaid, taſted like urine.

As the vomiting continued ſhe found relief from the ſoreneſs nnd ſwelling ſhe had felt for ſeveral days in the lower part of the abdomen.

She now thought herſelf much better, but the vomiting continued to return, more or leſs, every day, till the 14th of July, when Dr. Senter again ſaw her, and prevailed on her to ſubmit to the introduction of a catheter, by means of which he drew off about three pints of clear, but high-coloured, urine.

From this time, till December, ſhe continued with very little abatement of her complaints; and as ſhe could lie in no other poſition, was conſtantly ſupported in an arm chair, in a reclined poſture, with pillows under her hips.

During the whole of this period, whenever her water was omitted to be drawn off once in thirty or thirty-ſix hours at fartheſt, ſhe never failed, we are aſſured, to vomit it up. To aſ-

certain ſo extraordinary a fact, our author tells us he often viſited her about the time he knew ſhe muſt vomit if the catheter was not introduced; and after examining her bladder, and finding it full, hard, and tender, ſat by her till the vomiting returned, ſaved the water that ſhe brought up in this way, and on comparing it with what he drew off by means of the catheter, found it the ſame in every reſpect.

During the time her urine came off by vomiting, ſhe ſuffered, it ſeems, great anxiety and thirſt, and complained of a ſenſation of inverſion or turning up of ſomething (running, as ſhe expreſſed it) that appeared to tear her bowels.

In January, 1787, from ſome cauſe unknown, ſhe could not be relieved with the inſtrument, nor could ſhe vomit up her urine for ſeveral days; but at length it paſſed by the navel for three days ſucceſſively; after which the catheter was uſed with the ſame effect as before.

About the beginning of Auguſt a brick-coloured gravel began to paſs off through the catheter, and continued to be diſcharged in conſiderable quantity, whenever her urine was drawn off, till the beginning of November; at which time ſhe felt more diſtreſs than uſual, whenwhenever her urine came off by vomiting, and ſhe ſoon obſerved a gritty ſubſtance in her mouth. When our author was informed of this new phenomenon, he requeſted her to ſave the urine for his inſpection the next time ſhe vomited; and on comparing it with what he drew off, found it contained the ſame kind of gravel as that which paſſed the catheter.

From this period, to the ſummer of 1788, her complaints, he obſerves, continued much the ſame; but during that ſummer ſhe twice paſſed a ſmall quantity of urine through the urethra, each time in conſequence of being frightened. The hypogaſtrium became more tumid, and ſhe complained of great ſoreneſs about the bladder, even after it was evacuated; the bladder itſelf ſeemed to be much thickened, and the apparent inequality of its ſurface was ſo great, and the tumour ſometimes ſhifted ſo towards the right or left inguen, according as her body was moved, that our author ſuſpected the exiſtence of a ſtone.

Through the month of September her urine, we are told, could very rarely be drawn off; for upon the introduction of the catheter, a ſpaſm ſeized the urethra and neck of the bladder, ſo that although the inſtrument ſeemed to paſs high up into the bladder, not more than a gill of urine could be drawn off, before it ſtopped entirely, with a ſenſation of ſomething falling down againſt the cervix, which ſhe was confident was a ſtone; and early in the following month, Dr. Senter being able to introduce a ſound, readily met with a ſtone, which ſeemed to be of a ſmall ſize, and ſofter than urinary calculi commonly are.

She had at different ſeaſons of the year ſeveral ſmall abſceſſes on different parts of her body, but they did not appear to relieve her general complaints. She alſo voided at times (after ſhe had thrown up her urine) a bloody pus, of a coppery taſte. This purulent diſcharge, it is obſerved, was never expectorated by coughing, though ſhe had at times a dry cough, but was conſtantly brought up by vomiting.

In the ſpring of 1789 her urine began to paſs *per anum*, loaded with the ſame kind of gravel that had come away by the catheter. This diminiſhed but did not put a ſtop to her vomiting; for ſhe continued to throw up more or leſs gravel that way every week. This new courſe of her urine occaſioned a troubleſome diarrhœa and teneſmus, but ſhe felt leſs inconvenience from the ſtone in the bladder.

After the 13th of May her bladder never became ſo much diſtended with urine as it had been before; and the ſecretion of urine, as well as the formation of gravel, we are told, evidently diminiſhed in proportion to her loſs of ſtrength, and the increaſe of the diarrhœa. The menſes, which, during the whole of her illneſs, had returned at irregular periods, now entirely ceaſed. During the ſummer, the frequency of vomiting increaſed; ſhe had ſeveral convulſive fits after vomiting; became more and more emaciated, and hectical; and, at laſt, lethargic; and on the 11th of Auguſt, 1789, died.

The body was examined the day after her death, by Dr. Senter, in the preſence of Dr. Waterhouſe, of Cambridge, and Dr. Maſon, of Philadelphia, who, as well as ſeveral other reſpectable medical practitioners, had occaſionally viſited her in her life-time, and ſeen her vomit up both urine and gravel.

On diſſection, nothing was diſcovered that could throw any light on the nature of the diſeaſe.

In the thorax, the only morbid appearance was an adheſion of part of the right lobe of the lungs to the pleura.

In

In the abdomen, the omentum was found much wafted, and of a dark gangrenous colour; the ftomach alfo is defcribed as being in a gangrenous ftate, and containing 'a femi-purulent matter, of a fœtid fcent;' but the weather, we find, was very warm, and the body in an offenfive ftate, at the time the diffection was made. Nothing particularly worthy of notice was obferved in the ftate of the liver, gall-bladder, inteftines, kidneys, or ureters. The urinary bladder was alfo in its natural ftate, not in the leaft thickened, and contained no fand or gravel. The uterus contained about a drachm of thick, fœtid pus, but had no other appearance of difeafe; the Fallopian tubes were larger than ufual, and ftrung with feveral hydatids of the fize of a walnut; the corpora fimbriata had a gangrenous appearance; the ovaria were enlarged to the fize of a fmall hen's egg, and diftended with a clear limpid fluid.

To the preceding hiftory Dr. Senter has added many judicious remarks; and in his attempt to account for the phenomena of fo very uncommon a cafe, has not omitted to avail himfelf of the modern doctrine of the retrograde motion of the lymphatics, and of the opinions of thofe writers who have maintained the exiſtence of a direct communication between the alimentary canal and the urinary bladder.

There are many inſtances, he obſerves, in medical books, of ſudden and partially-increaſed actions of the veſſels of the human body; but he candidly acknowledges that his reading has furniſhed him with no fact ſimilar to the extraordinary one which is the ſubject of the paper before us *: that which he conſiders as

* There are, however, upon record, two caſes which exhibit a ſtriking analogy to that of Dr. Senter's patient; and although they may have been overlooked, or perhaps diſregarded on a ſuppoſition of their improbability, they muſt now become extremely intereſting by the tendency they have to corroborate the curious and extraordinary facts he has related. Both the caſes we allude to occur in the Hiſtory of the Academy of Sciences at Paris, and are as follows:

Caſe I. " M. Maraldi has communicated to the Aca-

" demy the following caſe, from a letter addreſſed to him

" by M. Marangoni, phyſician at Mantua:

" A Nun, of the Order of St. Francis, in the convent of

" St. Joſeph, at Mantua. aged thirty-five years, of a thin

" and delicate habit of body, and who had long been ſub-

" ject to hyſterical complaints, was attacked with pains,

" ſpaſms, and ſwelling of the abdomen, to which ſucceeded

" a violent and alarming ſuppreſſion of urine. Soon after

" this ſhe felt a pain, which ſhe deſcribed as aſcending from

" the lower part of the abdomen to her ſtomach; and ſhe

" vomited as coming the neareſt to it, is a caſe deſcribed by Dr. Percival, in the ſecond volume of his Eſſays, Medical and Experimental, (8vo, London,

" vomited a fluid which, without any difficulty, was
" known to be urine. This vomiting continued forty days,
" during which time the patient voided no urine by the
" uſual channel, unleſs the ſurgeon drew it off with a ca-
" theter, and even then the quantity ſcarcely amounted to
" an ounce a day. At the end of the forty days, the urine
" ſpontaneouſly reſumed its natural courſe, and in a day or
" two the patient found herſelf perfectly recovered. But
" the vomiting of urine returned, and at the end of twenty-
" ſeven days, the patient complained of very acute pain
" about the region of the pubis. Her ſurgeon was deſirous
" of relieving her by means of the catheter, but there was
" ſuch a contraction of the urethra, that he found it impoſ-
" ſible to introduce even a probe into the bladder. The
" vomiting of urine has continued, and what is remarka-
" ble, there is no appearance of food mixed with it, even
" when the vomiting takes place ſoon after her meals.
" When M. Marangoni wrote this account, the patient had
" been in this ſtate thirty-two days.

" This ſingular complaint would lead one to think there

" is an immediate though hitherto undiſcovered commu-

" nication between the ſtomach and the urinary bladder;

" but M. Marangoni and the celebrated Lanciſi are of a

" different opinion; they both of them think, that in caſes

" of this kind a ſuppreſſion of urine takes place in the kid-

" neys; that is to ſay, that the kidneys ceaſe to extract this

" fluid

London, 1773) of a woman who, after a ſpontaneous vomiting of ſeveral days, during which ſhe brought up three gallons of water, was entirely cured of a dropſy of the ovarium.

" fluid from the blood, and that in their ſtead the glands of " the ſtomach perform this function."

Caſe II. " M. Lemery is acquainted with a Monk, who, " for about eight years, has been ſubject to a periodical vo- " miting, the fits of which are as regular as thoſe of a quar- " tan ague. Five hours, or thereabouts, before the vomit- " ing begins he complains of violent pains in his kidneys. " The vomiting continues, with intervals, four or five " hours. What he vomits is of a dirty red colour. It is " almoſt entirely water, but has a ſtrong urinous ſmell, and " the patient has no doubt of its being really urine, as he " eats but very little, and drinks more than the uſual portion " of a Monk. He drinks only wine, the colour of which " agrees with that of the fluid he vomits. A few hours " after the vomiting he finds himſelf well, and remains ſo " till the next fit. He uſes a great deal of exerciſe, with- " out which he thinks he ſhould ſuffer more. It is a known " fact, that in nephritic pains, which are always occaſioned " by obſtructions of the kidneys, the patients are ſubject to " frequent vomiting, and that what they bring up ſmells " much of urine."——*See* Hiſtoire de l'Academie Royale des Sciences, Années 1715 & 1722. EDITOR.

CATALOGUE

